# Oligomerization and DNA binding of Ler, a master regulator of pathogenicity of enterohemorrhagic and enteropathogenic *Escherichia coli*

**DOI:** 10.1093/nar/gks846

**Published:** 2012-09-08

**Authors:** Jesús García, Tiago N. Cordeiro, María J. Prieto, Miquel Pons

**Affiliations:** ^1^Structural and Computational Biology, Institute for Research in Biomedicine, ^2^Department of Microbiology and ^3^Department of Organic Chemistry, University of Barcelona, Barcelona 08028, Spain

## Abstract

Ler is a DNA-binding, oligomerizable protein that regulates pathogenicity islands in enterohemorrhagic and enteropathogenic *Escherichia coli* strains. Ler counteracts the transcriptional silencing effect of H-NS, another oligomerizable nucleoid-associated protein. We studied the oligomerization of Ler in the absence and presence of DNA by atomic force microscopy. Ler forms compact particles with a multimodal size distribution corresponding to multiples of 3–5 units of Ler. DNA wraps around Ler particles that contain more than 15–16 Ler monomers. The resulting shortening of the DNA contour length is in agreement with previous measurements of the length of DNA protected by Ler in footprinting assays. We propose that the repetition unit corresponds to the number of monomers per turn of a tight helical Ler oligomer. While the repressor (H-NS) and anti-repressor (Ler) have similar DNA-binding domains, their oligomerization domains are unrelated. We suggest that the different oligomerization behavior of the two proteins explains the opposite results of their interaction with the same or proximal regions of DNA.

## INTRODUCTION

Enterohemorrhagic (EHEC) and enteropathogenic *Escherichia coli* (EPEC) strains are closely related human pathogens that are responsible for food-borne infection outbreaks and cause diarrhea by colonizing intestinal mucosa. EHEC infections can be followed by extra-intestinal complications, such as the hemolytic uremic syndrome, a common cause of renal failure in children (1). EHEC, EPEC and the mouse pathogen *Citrobacter rodentium* form a distinctive attaching-and-effacing (A/E) lesion in the intestinal epithelium of the host. Most of the genes responsible for the establishment of such lesions are localized in a pathogenic island termed the *l*ocus of *e*nterocyte *e*ffacement (LEE) (2).

The LEE island comprises five major polycistronic operons, *LEE1* to *LEE5*, and several minor operons and monocistronic genes. The *LEE1*, *LEE2* and *LEE3* operons mainly encode the membrane-spanning components of a type III secretion system, whereas most *LEE4* genes encode secreted translocator and effector proteins and the *LEE5* operon encodes proteins involved in intimate attachment to the host cell. Additional genes within the LEE region encode transcriptional regulators, effector proteins and chaperones.

The concerted regulation of LEE genes is essential for successful colonization of the host. Under non-permissive conditions, unwanted LEE transcription is silenced by the global repressor H-NS. After a transition to virulence-inducing conditions, expression of the primary positive regulator of LEE transcription, Ler (*L*EE-*e*ncoded *r*egulator), encoded by the first gene of the *LEE1* operon, is activated. Ler stimulates the expression of LEE genes by alleviating H-NS-mediated repression. Direct binding of Ler to defined sites within the regulatory promoter region of target genes disrupts the H-NS-dependent repressor nucleoprotein complex, thus releasing transcription (2).

In addition to the LEE genes, there is also evidence that Ler acts as a positive regulator of numerous horizontally acquired genes that lie outside the LEE region (3–5). However, Ler does not upregulate other H-NS-silenced operons, such as *bgl* (6) and *proU* (3). This observation suggests that Ler is not a general antagonist of H-NS, but a specific activator of virulence genes acquired by horizontal transfer in A/E pathogens.

Both H-NS and Ler form oligomers and have a modular structure that comprises an N-terminal domain responsible for oligomerization and a C*-*terminal DNA binding domain. The DNA-binding domains of Ler and H-NS are highly related and they have recently been shown to recognize similar features in the DNA minor groove (7). Consistently, H-NS and Ler bind to overlapping DNA regions, although the former acts as a repressor while the latter is an activator of transcription (2). The functional opposite effects of Ler and H-NS may originate from the distinct oligomer types formed by these two proteins. A recent electron microscopy study has shown that Ler adopts a nucleoprotein structure distinct to the characteristic elongated bridges formed by H-NS. Ler oligomers were shown to have a globular appearance (8). Atomic force microscopy (AFM) offers supplementary quantitative information about the volume of the particles formed by Ler under a range of conditions and their effect on DNA. Our findings support the notion that DNA wraps around Ler oligomers.

## MATERIALS AND METHODS

### DNA substrates and proteins

The 1192-bp DNA fragment (Lee1192) was amplified by polymerase chain reaction (PCR) from EHEC strain 0157:H7 using forward (5′-GCC AGC GGC CGC TAA CGA GGT ATG CTC ATC AAC G-3′) and reverse (5′-GCG AGC GGC CGC ATT AAT AAA AGT AAA GCT GTC G-3′) primers. The PCR product was gel-purified in a 1% (w/v) agarose gel, excised, and eluted using the QIAEX II gel extraction kit (QIAGEN). The DNA concentration was determined from the absorption at 260 nm.

Ler is a regulator of horizontally transferred genes. We used the commercially available pBR322 plasmid (Inspiralis), widely used in protein-binding studies, as an example of horizontally transferred DNA. A relaxed form was used to avoid the potential problems of measuring the contour length of self-crossing segments of DNA.

For the insertion of amino acids GAGGDGGSG at position 73, two Ler fragments were generated by PCR using the following primer pairs: LerNcoI (5′-CCT G*CC ATG G*AA AAT AAT TCA CAT ACA ACA AGT C-3′) and LerNarIr (5′-CGC T*GG CGC C*TT TCG ATG AGT TCC GGC GAG C-3′); LerNarIf (5′-GGC T*GG CGC C*GG TGG TGA TGG CGG TAG CGG CGG TGT TTA CTA CCG CAA TGA AGA AG-3′) and LerXhoI (5′-GCG C*CT CGA G*TC ATT CTT CTT CAG TGT CCT TCA CAA G-3′) (the restriction sites used in cloning are italicized) and a Ler-encoding plasmid (7) as template. The PCR-amplified fragments were digested with *Nar*I, ligated and subjected to a second amplification using LerNcoI and LerXhoI as primers. The restriction sites *Nco*I and *Xho*I were used to clone the resulting DNA fragment into the pHAT2 plasmid.

The expression and purification procedure for Ler (residues 3–116) and full-length H-NS has been described previously (7). The mass predicted from the Ler amino acid sequence is 14.3 kDa and was confirmed by mass spectroscopy.

### AFM imaging and data analysis

DNA samples were diluted in deposition buffer (10 mM Tris, 100 mM NaCl, 7 mM MgCl_2_ pH 7.5) to a final concentration of 3.5 ng/μl. For imaging isolated Ler samples, Ler was diluted in deposition buffer from stock (8–10 μM) to a final concentration of 0.2 μM. Ler–DNA complexes were formed in deposition buffer by mixing 57 ng of Ler with either 40 ng of Lee1192 or 70 ng of pBR322 in a total volume of 20 μl. This corresponds to a Ler final concentration of 0.2 μM. pBR322, H-NS and Ler were mixed in deposition buffer containing 1 mM *tris*(2-carboxiethyl)-phosphine (TCEP) to a final concentration of 3.5 ng/µl (pBR322), 0.4 µM (H-NS) and 0.2 µM (Ler).

Protein and DNA solutions were incubated at room temperature for 15 min and deposited (3 μl) onto freshly cleaved mica. After incubation for 1 min, the surface was rinsed with Milli-Q (Millipore) water and dried under a gentle flux of nitrogen gas.

AFM imaging was performed with a Multimode 8 atomic force microscope attached to a Nanoscope V electronics (Bruker, Santa Barbara, CA, USA) operating in Peak Force mode at room temperature. Image resolution was set at 640 × 640 pixels, scan rate at 1 Hz and the scan size of recorded images was 2 × 2 μm. AFM probes used for these experiments have triangular silicon nitride cantilevers with silicon oxide pyramids (SNL-10, Bruker). The nominal spring constant was 0.35 nN/nm and the tip radius ∼5 nm.

Nanoscope image processing software was used to flatten all AFM images prior to analysis. The DNA contour lengths and end-to-end distances were manually measured by using Image J software (version 1.46f). Measurements were performed only on those DNA molecules that were completely visible in the image and in which the shape was not ambiguous. The contour length of Lee1192 molecules with end-bound Ler could not be unambiguously measured and were omitted from the analysis.

The apparent volume of proteins was estimated using the bearing analysis function of the Nanoscope software. For each spot, the bearing volume was measured from the top to the 85% of the total height. AFM volumes can be translated into protein molecular weights by calibrating the instrument with proteins of known molecular weight to derive a linear relationship (Supplementary Figure S1). For our setup, we thus found *V = *4.2 × (MW)* − *126.5, where *V* is the AFM volume and MW is the molecular weight.

## RESULTS

### Ler–DNA complexes imaged by AFM

Samples of isolated Ler oligomers, free DNA molecules and Ler–DNA complexes deposited on freshly cleaved mica in Mg^2+^-containing buffer were imaged by AFM in air. Linear and circular DNA molecules were studied. A 1192-bp linear fragment (Lee1192) containing nucleotides −591 to +581 relative to the *LEE2* transcriptional start site was generated by PCR. This fragment includes a fragment of 121 bp (*LEE2* positions −221 to −101), which was found to be protected by Ler from DNAse attack in footprinting experiments (9). The circular DNA molecule was the relaxed form of plasmid pBR322 (4361 bp). The use of circular DNA molecules prevents the presence of free ends, which have a high tendency to bind Ler particles *in vitro*. The AFM images of Ler complexes formed with circular and non-terminal regions of linear DNA molecules were found to be very similar (discussed later).

**Figure 1.**
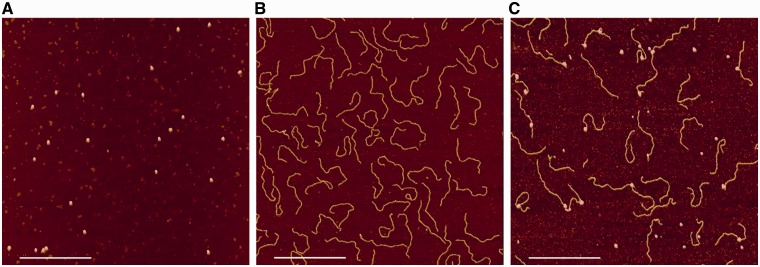
AFM imaging of Ler, Lee1192 and Ler/Lee1192 samples. Typical AFM images of isolated Ler (0.2 µM) (**A**) and the linear 1192-bp DNA fragment (Lee1192, 2 ng/µl) in the absence (**B**) and in the presence of 0.2 µM Ler (**C**). The white bar indicates 500 nm.

Figure 1 shows AFM images of purified Ler, isolated Lee1192 DNA and Ler/Lee1192 mixtures. Visual inspection of the images revealed that Ler, in the absence and in the presence of DNA, appeared as globular particles, similar to those reported in a recent electron microscopy study (8).

**Figure 2.**
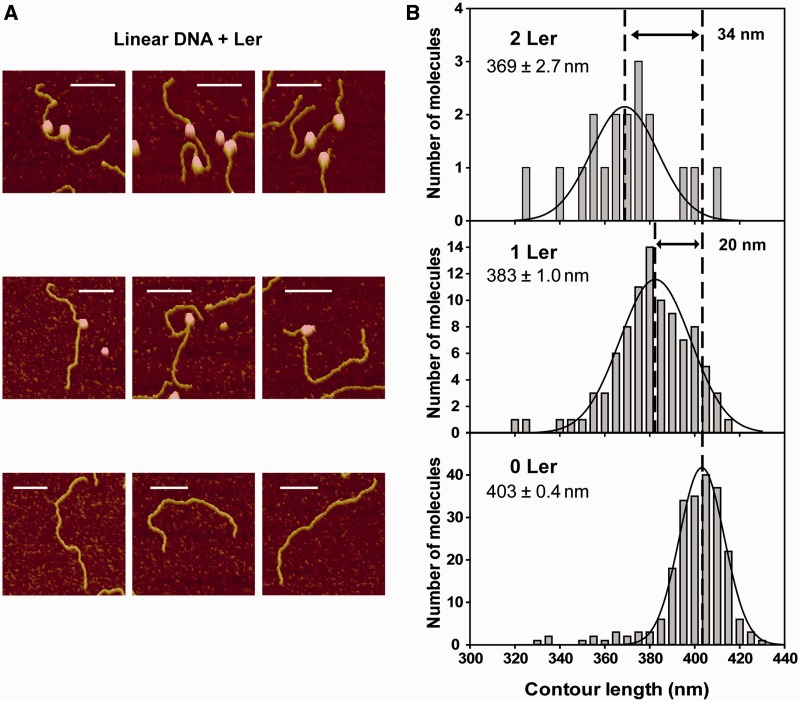
Shortening of linear DNA molecules induced by Ler. (**A**) Magnified AFM images of free Lee1192 molecules (bottom images) and Lee1192 molecules bound to one (middle images) or two (top images) Ler particles found in the same deposition. The white bar indicates 100 nm. (**B**) Contour length distribution of unbound Lee1192 (0 Ler, *N *= 220) and Lee1192 molecules bound to one (1 Ler, *N* = 93) and two (2 Ler, *N* = 18) Ler particles measured in the presence of Ler. The curves represent Gaussian fitting of the distributions. The mean ( ± standard error of the mean) are included.

In most of the Ler–Lee1192 complexes imaged by AFM, a sharp DNA bend was observed at the position where Ler particles were bound (Figures 1C and 2A). A figure showing the frequency distribution of the Ler-induced bending angles is presented as supplementary material (Supplementary Figure S2). To quantify the effect of the attachment of these particles to DNA molecules, we measured the contour length of free and Ler-bound DNA molecules that could be unambiguously traced from end to end in the images. In total, 220 unbound and 111 Ler-bound Lee1192 molecules were measured. Of the latter, 93 were bound to one Ler particle while 18 had two Ler particles attached. Some representative examples are shown in Figure 2A and the contour length distributions are shown in Figure 2B.

The contour length of free Lee1192 shows a Gaussian distribution centered at 403 ± 0.4 nm. This value corresponds to 2.96 bp/nm or an axial base pair rise of 0.34 nm/bp, in agreement with the values reported in other AFM studies (10,11). The analysis of free DNA molecules present in the images of samples that also contained Ler–DNA complexes was used as a control of the deposition conditions applied. It has previously been argued that structural information obtained from AFM is reliable only for samples that are deposited in such a way that they can freely equilibrate on the mica surface (2D-equilibration) (12). The alternative, undesirable situation corresponds to molecules kinetically trapped on the mica surface (2D-projection). The two scenarios can be distinguished by experimentally measuring the mean square end-to-end distances (*<R^2^>*) and comparing them with the predictions for a DNA molecule of a given contour length (*L*) and persistence length (*P*) based on the two alternative deposition modes given by the following equations: <*R^2^*>_2D_ = 4*PL* [1 − 2*P*/*L* (1 − *e^−^^L/^^2^^P^*)] and <*R*^2^>_proj_ = 4*PL*/3 [1 − 2*P*/*L* (1 − *e^−^^L/P^*)] (13).

The *<R^2^>* predictions for Lee1192 molecules based on the experimental contour length of 403 nm and assuming the standard value of 53 nm for the DNA persistence length were 63 465 nm^2^ and 24 735 nm^2^ for <*R^2^*>_2D_ and <*R^2^*>_proj_, respectively. The experimentally observed value was 60 690 nm^2^, thus confirming that the DNA samples were not kinetically trapped.

### Changes in DNA contour length induced by Ler binding

The DNA contour length for Ler-bound DNA molecules was measured tracing the DNA path from end to end passing through the center of the protein (14). The contour length distributions obtained for DNA molecules bound to one or two Ler particles were centered at 383 ± 1.0 nm and 369 ± 2.7 nm, respectively, (Figure 2) and showed a reduction with respect to free DNA molecules (403 ± 0.4 nm). The differences are statistically significant (*P* < 0.0001). The larger dispersion of the contour lengths of Ler-bound DNA may result from the fact that DNA-bound oligomers of different sizes were not differentiated in these measurements (discussed later). The mean of the contour length distributions was reduced by ∼20 and 34 nm with respect to free DNA when one or two Ler particles were bound, respectively.

The contour length of DNA molecules with Ler particles bound to their ends could not be unambiguously measured and these molecules were omitted from the analysis. Binding to the DNA ends was also observed with linear fragments of several sequences (result not shown) and can be considered an unwanted side effect of the use of linear DNA fragments. This effect that may be related with the possibility of forming DNA–protein interactions without the energetic cost associated to DNA distortion can be avoided by using circular DNA.

**Figure 3.**
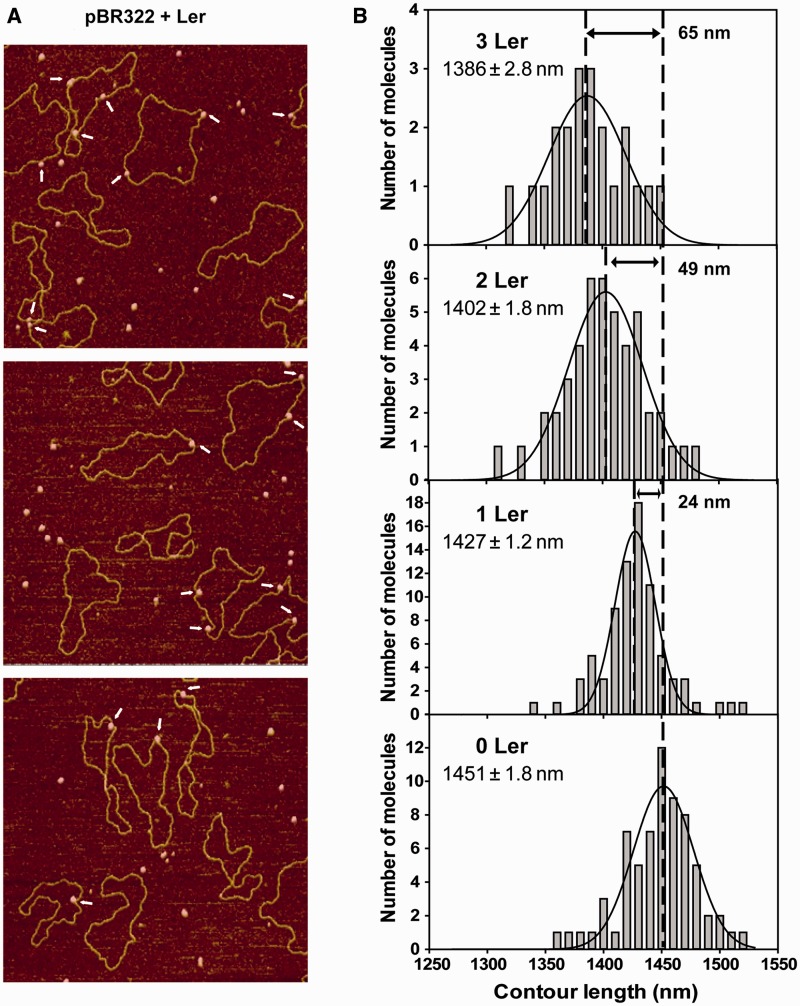
Shortening of pBR322 molecules induced by Ler. (**A**) Typical AFM images of the relaxed form of pBR322 plasmid in the presence of Ler. Shown are 1.5 × 1.5 µm areas from 2.0 × 2.0 µm images. Arrows point to Ler–DNA complexes. (**B**) Contour length distribution of unbound pBR322 (0 Ler, *N* = 67) and pBR322 bound to one (1 Ler, *N* = 79), two (2 Ler, *N* = 46) and three (3 Ler, *N* = 21) Ler particles measured in the presence of Ler. The curves represent Gaussian fitting of the distributions. The mean (± standard error of the mean) are included.

Ler particles bound to relaxed pBR322 plasmid (Figure 3). The longer length of pBR322 compared with Lee1192, as well as the absence of unwanted end-binding effects, allowed the measurement of complexes containing a larger number of simultaneously bound Ler particles. As in the case of linear Lee1192, sharp bends were often observed at the sites where Ler particles were bound (Figure 3, Supplementary Figure S2).

The contour lengths of free and Ler-bound pBR322 molecules in the same deposition were measured (Figure 3B). The distribution of contour lengths obtained from 67 protein-free pBR322 molecules had a mean of 1451 ± 1.8 nm, giving a base pair rise of 0.33 nm/bp, similar to that observed in the linear fragment.

A significant reduction of contour length was observed for DNA molecules with attached Ler particles with respect to free pBR322 (*P* < 0.0001). The average contour length was reduced by ∼24, 49 and 65 nm, respectively, in the presence of one (*N* = 79), two (*N* = 46) or three (*N* = 21) Ler particles bound to the DNA (Figure 3B).

**Figure 4.**
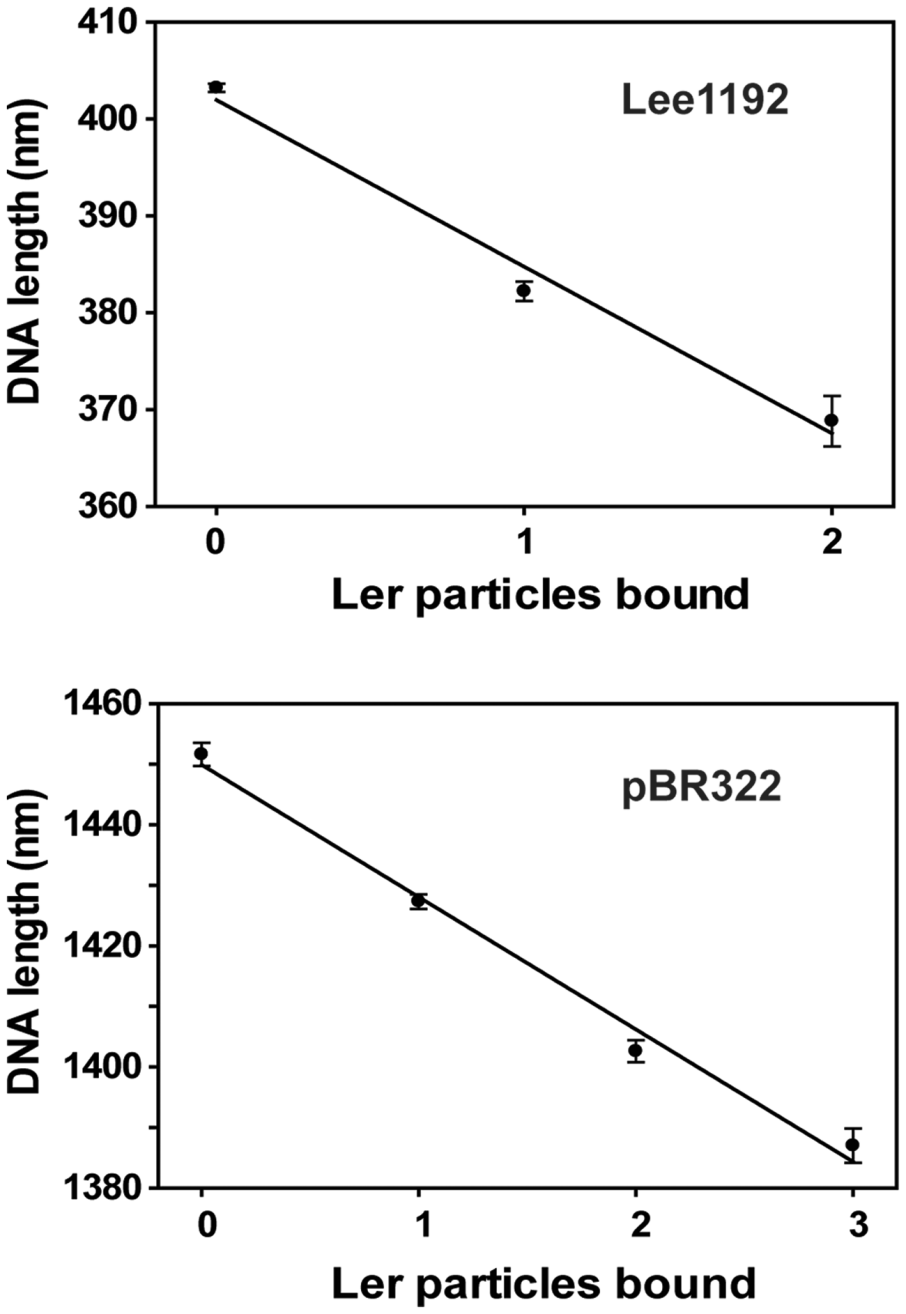
DNA compaction induced by Ler. Plots of DNA contour length (mean ± standard error of the mean) obtained for Lee1192 (top panel) and pBR322 (bottom panel) molecules versus the number of bound Ler particles. The slope of the regression line, 17.2 ± 2.0 nm (Lee1192, *R*^2 ^= 0.98) and 21.9 ± 1.5 nm (pBR322, *R*^2 ^= 0.99) represents the average DNA compaction induced by each Ler particle.

Figure 4 shows a plot of the contour lengths of Lee1192 and pBR322, as measured by AFM, as a function of the number of bound Ler particles. A linear dependency was observed in both cases with a slope that represents an average shortening of 17.2 ± 2.0 and 21.9 ± 1.5 nm per Ler particle bound to Lee1192 and pBR322, respectively.

### Size distribution of Ler particles

All Ler particles look globular at the AFM resolution. However, a detailed analysis of the distribution of the particle sizes that can be quantified by analysis of the AFM images and the compaction they induce in DNA, also measured by AFM, provide additional constrains about their actual shape, which is consistent with short cylinders of constant diameter (discussed later). Although apparent volumes measured by AFM deviate significantly from real volumes as a result of tip-sample interactions, several authors have established that there is a linear dependence between the molecular weight of a protein and its volume measured by AFM (11,15–20).

**Figure 5.**
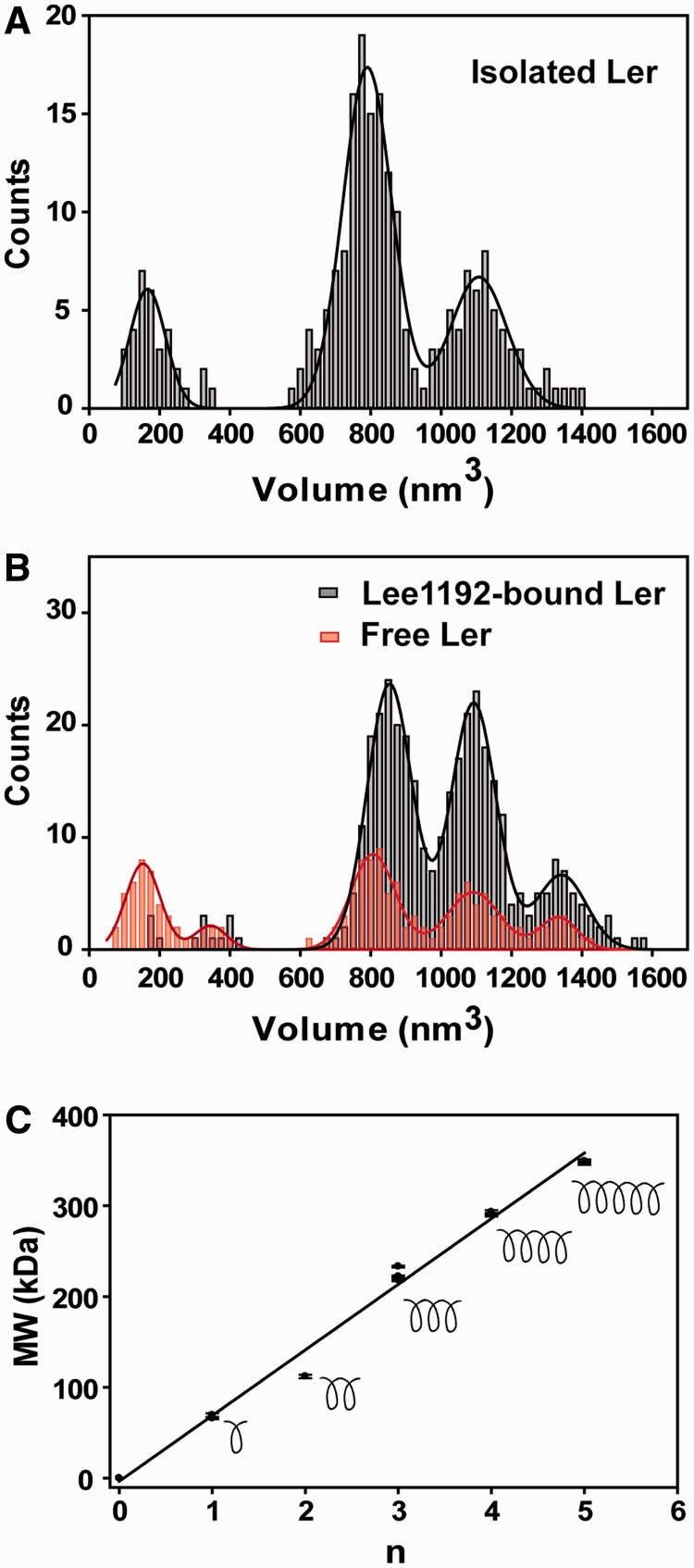
AFM analysis of Ler volume. Distribution histogram of apparent volumes of Ler particles measured in (**A**) isolated Ler (0.2 µM. See Figure 1A for a representative image). (**B**) Ler (0.2 µM) in the presence of linear DNA (Lee1192, 2 ng/µl). See Figures 1C and 2A for representative images. The volume distributions of unbound (red bars) and DNA-bound (black bars) Ler particles are shown. (**C**) Linear fitting showing that the apparent molecular mass of Ler particles corresponds to multiples of a basic oligomeric form, which we interpret as turns of a helical arrangement of Ler monomers (see text for details).

The volume of Ler particles was measured in AFM images of samples of isolated Ler (0.2 µM) and for Ler/Lee1192 mixtures (0.2 µM Ler, 2 ng/µl DNA). Figure 5A shows the distribution of volumes of 217 Ler particles deposited in the absence of DNA. A multimodal distribution was clearly observed with three main peaks, which were fitted to Gaussian distributions with means and standard errors of 166 ± 8, 790 ± 3 and 1107 ± 9 nm^3^, respectively (Supplementary Table S1). A small number of particles with a volume of ∼340 nm^3^ were also observed.

A similar analysis was performed in AFM images of samples deposited after incubation of Ler with Lee1192. These images contained both free and DNA-bound Ler particles and the distributions of the two forms were analysed independently.

The volume distribution of 153 free Ler particles observed in samples previously incubated with DNA is shown in Figure 5B (red bars). A multimodal distribution was also observed in this case but with five clearly defined peaks centered at 153 ± 3, 344 ± 5, 806 ± 3, 1092 ± 5 and 1333 ± 8 nm^3^ (mean ± standard error of the mean) (Supplementary Table S1). The 806 and 1093 nm^3^ peaks closely matched those observed in Ler particles not incubated with DNA, although their relative populations differed substantially. The small number of particles observed around 340 nm^3^ in the absence of DNA could correspond to the peak observed at 344 nm^3^ in the presence of DNA. The additional peak at higher volume present only in DNA-incubated samples was observed for both free and DNA-bound Ler particles (discussed later).

To rule out the trivial possibility that the Ler size distributions differ from experiment to experiment, we compared multiple independent depositions of DNA-free Ler particles and found no statistically significant differences (Kolmogorov–Smirnov test). In contrast, the size distributions of Ler particles in the presence and in the absence of DNA are significantly distinct (*P* < 0.004).

The distribution of the measured volumes for Lee1192-bound Ler (355 particles) is shown in Figure 5B (black bars). A three-peaked distribution was observed with mean values of 853 ± 4, 1093 ± 3 and 1341 ± 9 nm^3^ obtained by Gaussian fitting. Although complexes formed by small Ler oligomeric assemblies were occasionally observed (14 of 355, ∼3.9%), DNA complexes with large Ler particles were clearly favored.

The apparent molecular weight of Ler particles was estimated from the calibration obtained by measuring a set of reference proteins under the same experimental conditions. As previously described (15,18–20), a linear relationship between the measured volumes and the corresponding molecular weights of the standard proteins was obtained (Supplementary Figure S1). Using this calibration, the apparent molecular weight of the center of the peaks in the distributions of free Ler particles were 67, 112, 220, 291 and 347 kDa, which correspond to ratios with respect to the nominal mass of the Ler monomer (14.3 kDa) of 4.7, 7.8, 15.4, 20.3 and 24.3.

An independent analysis using size exclusion chromatography of a more concentrated (8 µM) sample gave a broad peak with an apparent molecular weight of 355 kDa, which would correspond to 24–25 copies of Ler, although the presence of smaller oligomers in the tail of the peak cannot be ruled out (Supplementary Figure S3).

Very similar apparent molecular weights were observed in free and DNA-bound Ler particles, especially for the larger species. The apparent volume of the first peak in the distribution of DNA-bound particles was slightly larger than the equivalent peak from the free particle distribution (853 nm^3^ vs. 790–806 nm^3^). This small difference may arise from the contribution of DNA to the measured volume. This effect was negligible in the larger particles.

## DISCUSSION

A detailed AFM analysis of the particles formed by free Ler and their complexes with DNA reveals new details on the oligomerization properties of Ler and its interaction with DNA.

The size distribution of the Ler particles observed by AFM shows a regular pattern with peaks that correspond to molecular weights that are approximately integer multiples of a fixed value. Figure 5C shows that the apparent molecular mass (MW) of Ler particles follows a linear equation (regression coefficient 0.99) of the form MW = *kn* + *ε*, where *n* are integers from 0 to 5, and *k* and *ε* are adjustable parameters. The slope is 72.3 ± 2.4 kDa, which corresponds to 5.05 units of Ler.

Previous experiments using cross-linking and tag-capture experiments from mixtures of His-tagged and non-tagged mixtures carried out in the Rosenshine group (21) showed that in Ler oligomers each Ler molecule preferentially interacts with two other monomers (N. Chan, PhD thesis; I. Rosenshine, personal communication), thereby suggesting an infinite linear arrangement. A similar situation can be found in the case of H-NS. Although truncated forms form discrete dimers, the entire protein assembles into potentially infinite linear oligomer by alternating head-to-head and tail-to-tail dimerization units (22). The periodic size distribution of Ler particles observed by AFM could also be explained by a helical arrangement of Ler monomers in which certain oligomer lengths are preferred. An obvious hypothesis is that the observed particles correspond to the oligomers that adopt full helical turns. Under this hypothesis, the observed slope would correspond to a helical arrangement with five Ler monomers per turn. However, 3 and 4 monomers per turn could also reasonably explain the data, assuming that some of the intermediate values of *n* in the series are not observed (Supplementary Figure S4).

The AFM-determined molecular weight of the most abundant Ler particles closely matches the size of the smaller oligomers (75–200 kDa) described by Mellies *et al.* using size exclusion with in-line multi-angle laser light scattering (SEC-MALS). Our AFM images also revealed the presence of rare (less than 1%) large Ler assemblies of ∼10 000 nm^3^, which were not included in the analysis of size distributions. Assuming that the linear dependence between the AFM-measured volume and molecular weight is still applicable to this protein size, the volume of these large Ler assemblies would be consistent with a multimer of about 2400 kDa, in agreement with the higher aggregates observed by SEC-MALS (8).

The population of the different classes of Ler particles observed by AFM is clearly affected by incubation with DNA. Samples incubated with DNA show larger particles, thereby suggesting that DNA binding facilitates their assembly. However, once formed, the larger particles are stable even after dissociating from DNA, as shown by the similar size distribution of free and DNA-bound Ler particles in samples incubated with DNA. On the other hand, when comparing the distributions of DNA-bound and free particles observed in the same DNA containing sample, it is apparent that cooperative binding to a minimum of ∼15 Ler molecules per particle is required to form Ler–DNA complexes that are stable enough to be observed by AFM. In addition to cooperative binding, a lower limit to the size of Ler particles that can bind DNA may be determined by the maximum possible bending of DNA to wrap around the particle (discussed later).

The interaction with Ler particles resulted in a linear decrease in the DNA contour length of ∼20 nm per Ler particle and was similar for linear (Lee1192; 17.2 ± 2.0 nm) and circular (pBR322; 21.9 ± 1.5 nm) DNA. The reduction in contour length is consistent with DNA wrapping around Ler oligomers, as previously proposed by Mellies on the basis of the comparison of the diameter of the complexes observed by electron microscopy with previous footprinting experiments (8). DNA contour lengths of Ler–Lee1192 complexes were measured assuming that DNA passed through the center of the Ler particle. The length of the DNA hidden by Ler particles *<L*_H_*>* can be estimated by *<L*_H_*>* = *<L*_F_*>* − *<L*_V_*>* = *<L*_F_*>* − *(<L*_B_*>* − *<L*_Ler_*>*), where *<L*_F_*>* and <*L*_B_> are the mean values of the length of free and Ler-bound DNA, respectively, *<L*_V_*>* is the visible DNA length not interacting with Ler, and *<L*_Ler_*>* is the average diameter of Ler particles (21 nm). The length of DNA hidden by Ler estimated in this way ranged from 38 to 43 nm, which corresponds to 112 to 127 bp. Although this may represent an overestimation of the occluded DNA by Ler as a result of tip broadening effects, these values are in good agreement with the 117 to 150 bp DNA fragments protected by Ler from DNAse digestion in footprinting experiments (6,9,23,24).

Ler binding relieves the repression caused by H-NS. The two proteins share similar DNA binding sites and a common indirect readout mechanism (7). They also share similar domain architectures, with an oligomerization domain connected to the DNA-binding domain through a flexible connector. The X-ray structure of the oligomerization domain of H-NS showed an arrangement of H-NS units alternating head-to-head and tail-to-tail contacts to form a potentially infinite superhelical extended polymer (22). The oligomerization domains of the two proteins show no apparent sequence homology and the macroscopic appearance of the oligomers formed by the two proteins is apparently quite different. However, the analysis of Ler oligomers suggest that H-NS and Ler also share a similar oligomerization architecture based on a linear assembly of monomers that fold into a helical conformation, although the pitch is much smaller for Ler than for H-NS leading to compact, globular shapes in the case of Ler but extended forms for H-NS. Bound DNA must adopt very different shapes to contact the DNA-binding domains attached to the oligomers of Ler or H-NS. The superhelical H-NS oligomer has been suggested to form a scaffold to which two double stranded DNA segments adopting a superhelical pitch matching that of the protein is attached (22). In the case of Ler, DNA molecules cannot follow the superhelical pitch of the protein oligomers but, instead, wrap around Ler particles. The length of DNA needed to wrap around Ler particles is compatible with the known bending rigidity of DNA segments. Although DNA can bind to Ler particles formed by various numbers of monomers, the average DNA compaction per Ler particle is independent of the particle size: Ler particles containing 15–16 and 24–25 Ler monomers caused average DNA compaction of 19.1 ± 2.1 nm and 19.8 ± 2.3 nm. This observation suggests Ler particles are cylinders of constant diameter with DNA wrapping around, although the resolution of the AFM images does not allow a direct visualization of its exact shape. A minimum length of the Ler oligomer, allowing at least three complete turns of the helical Ler oligomer seems to be required for DNA binding. The exact matching between DNA-bending and the diameter of the Ler particles should be dependent on the length and flexibility of the connector linking the Ler DNA-binding and oligomerization domains. This effect was checked by experimentally increasing the length of the connector with the insertion of a nine amino acid segment. The longer, more flexible connector allowed for a looser fit between DNA and the Ler particles and resulted in a significantly lower apparent DNA bending (Supplementary Figure S5).

The similar architecture of H-NS and Ler and their similar DNA recognition properties contrast with their opposite functional roles as repressor and anti-repressor, respectively of a number of genes (2,4,6,9,24,25). The distinct oligomers formed by Ler and H-NS most probably play a key role in their competing functions.

**Figure 6.**
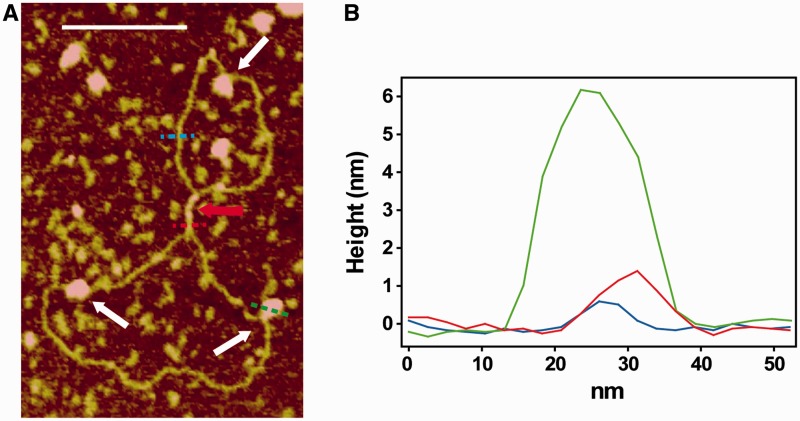
Co-existing Ler– and H-NS–DNA complexes. (**A**) Representative AFM image of pBR322 (3.5 ng/µl) in the presence of H-NS (0.4 µM) and Ler (0.2 µM). Nucleoprotein complexes formed by H-NS and Ler are indicated by red and white arrows, respectively. The white bar indicates 175 nm. (**B**) Cross-sections along the positions highlighted by the dashed lines in (A) that correspond to free DNA (blue), an H-NS–DNA complex (red) and a Ler–DNA complex (green).

Many promoters are repressed by H-NS binding simultaneously to sites located upstream and downstream of the promoter. In some cases, Ler could relieve H-NS repression caused by DNA looping by displacing H-NS from one of the sites but other situations may be possible. Mutual exclusion between Ler and H-NS may be mediated by DNA distortions associated to DNA wrapping and is clearly seen in Figure 6 showing AFM images of samples containing DNA in the presence of both Ler and H-NS. Other proteins releasing H-NS-mediated repression have been shown to induce changes in DNA topology (26–29).

The results reported here reveal details of the modular architecture of Ler oligomers and provide evidence of DNA wrapping around Ler particles. Further efforts will be directed toward the detailed structural characterization of the basic units on which Ler oligomers are based. These may provide relevant insight, possibly pointing to strategies to interfere with Ler-based activation in pathogenic *E. coli* strains.

## SUPPLEMENTARY DATA

Supplementary Data are available at NAR Online: Supplementary Table 1 and Supplementary Figures 1–5.

## FUNDING

Spanish Ministry of Research, Development and Innovation; the European Fund for Regional Development [BIO2010-15683]; Generalitat de Catalunya [2009SGR1352]; European Community’s Seventh Framework Program [Bio-NMR contract 261863]. Funding for open access charge: Generalitat de Catalunya [2009SGR1352].

*Conflict of interest statement*. None declared.

## Supplementary Material

Supplementary Data
