# Supplementary material for: Oligomerization and DNA binding of Ler, a master regulator of pathogenicity of enterohemorrhagic and enteropathogenic Escherichia coli
Source: Nucleic Acids Res. 2012 Sep 8;40(20):10254–62. doi: 10.1093/nar/gks846 (PMC3488262; doi:10.1093/nar/gks846)
Supplement: Supplementary Data [file supp_40_20_10254__index.html]

Oligomerization and DNA binding of Ler, a master regulator of pathogenicity of enterohemorrhagic and enteropathogenic Escherichia coli — Oligomerization and DNA binding of Ler, a master regulator of pathogenicity of enterohemorrhagic and enteropathogenic Escherichia coli — Supplementary Data 

# Oligomerization and DNA binding of Ler, a master regulator of pathogenicity of enterohemorrhagic and enteropathogenic *Escherichia coli*

## Supplementary Data

files

**Files in this Data Supplement:**

- Supplementary Data - pdf file
