# Supplementary material for: Oligomerization and DNA binding of Ler, a master regulator of pathogenicity of enterohemorrhagic and enteropathogenic Escherichia coli
Source: Nucleic Acids Res. 2012 Sep 8;40(20):10254–62. doi: 10.1093/nar/gks846 (PMC3488262; doi:10.1093/nar/gks846)
Supplement: Supplementary Data [file supp_gks846_nar-01643-v-2012-File008.pdf]

Oligomerization and DNA binding of Ler, a master regulator of pathogenicity of enterohemorrhagic and enteropathogenic *Escherichia coli*

Supporting Information

Jesús García[1,]*, Tiago N. Cordeiro[1], María J. Prieto[2] and Miquel Pons[1,3,]*

[1] Structural and Computational Biology, Institute for Research in Biomedicine, Barcelona, 08028, Spain

[2] Department of Microbiology, University of Barcelona, Barcelona, 08028, Spain

[3] Department of Organic Chemistry, University of Barcelona, Barcelona, 08028, Spain

*Corresponding author: jesus.garcia@irbbarcelona.org (JG); mpons@ub.edu (MP)

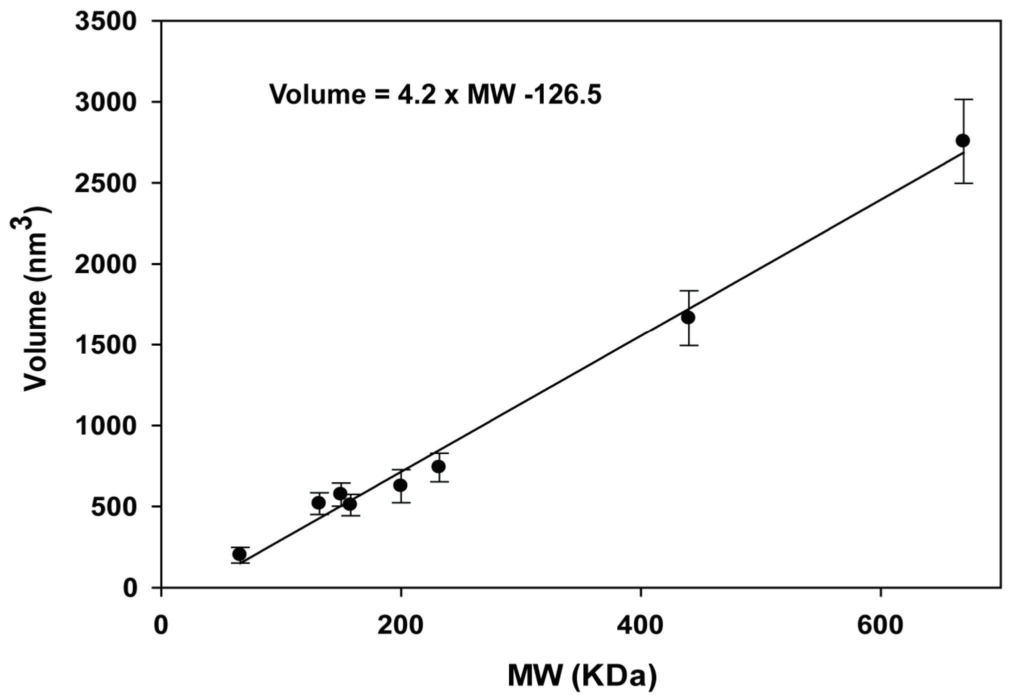

Fig. S1. Correlation between AFM-volume and molecular weight for protein standards. Average AFM volume (measured in N particles) plotted versus molecular weight for: Bovine serum albumin (monomeric, 66 kDa, N=40), Bovine serum albumin (dimeric, 132 kDa, N=30), Alcohol dehydrogenase (150 kDa, N=50), Aldolase (158 kDa, N=50), Amylase (200 kDa, N=25), Catalase (232 kDa, N=200), Ferritin (440 kDa, N=100) and Thyroglobulin (669 kDa, N=30). Sample preparation and imaging conditions were equivalent to those used for Ler imaging. The line represents the fit of the data to the following equation: *Volume = 4.2 x MW – 126.5*, where *MW* is the molecular weight ($R^2 = 0.99$).

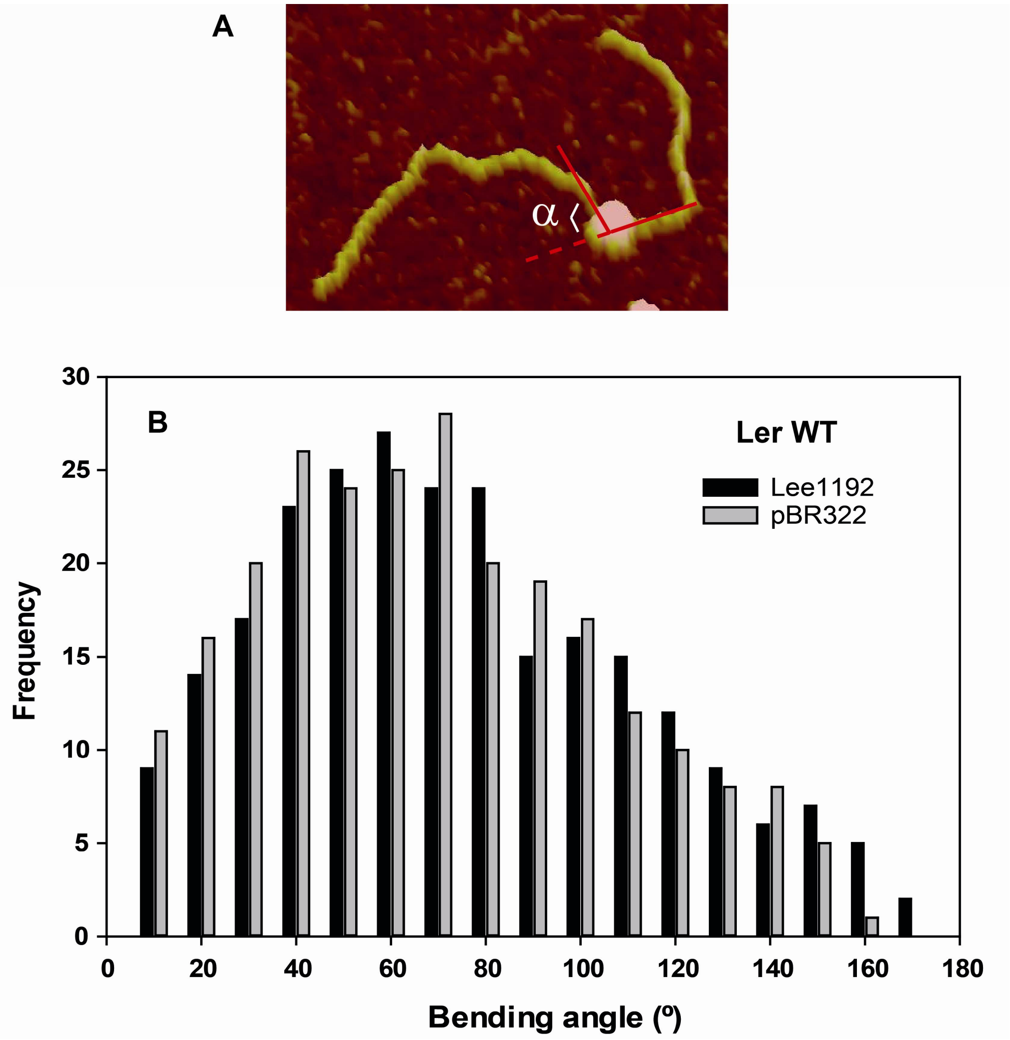

Fig. S2. Ler-induced DNA bending. (**A**) DNA-Ler complex showing the definition of the apparent bend angle (α). (**B**) Histogram of the Ler-induced bending-angle distributions in sets of 250 Ler/Lee1192 complexes and 250 Ler/pBR322 complexes.

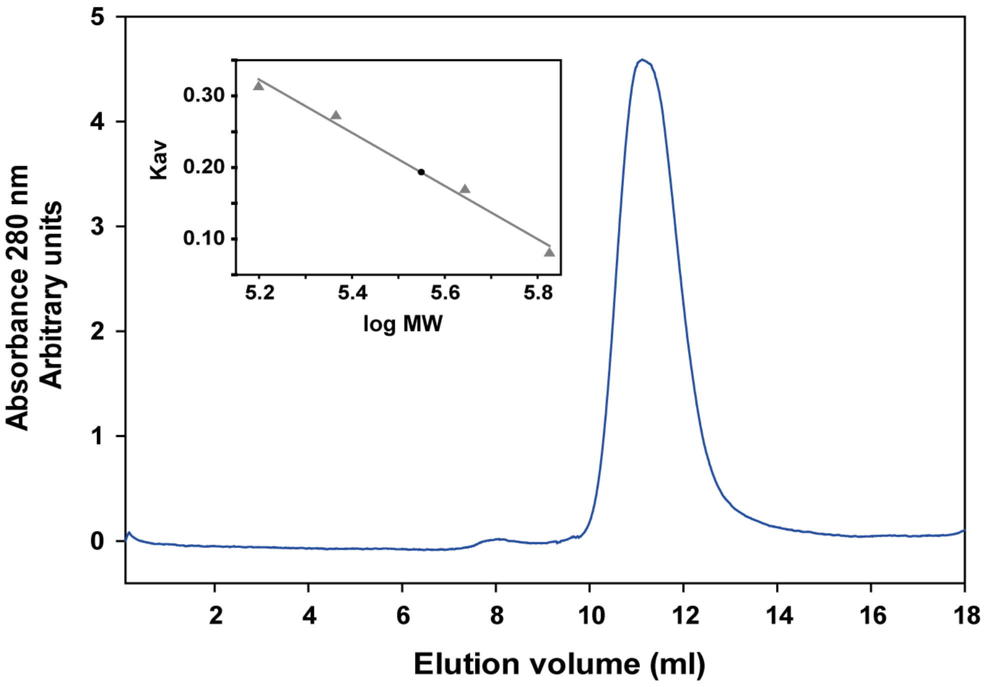

Fig. S3. Size exclusion chromatography of Ler. Elution profile of Ler (8 µM) on a Superdex200 10/300 GL column (GE Healthcare). Shown in the inset is the calibration curve using the following standard proteins (grey triangles): Aldolase (158 kDa), Catalase (232 kDa), Ferritin (440 kDa) and Thyroglobulin (669 kDa). Elution data were analyzed according to the formula *Kav = (Ve-Vo)/(Vt-Vo)*, where *Ve* is the elution volume. The total volume (*Vt*) of the column is 24 ml, and the void volume (*Vo*) of the column is 8.1 ml, as determined by blue dextran 2000. From this calibration, the log MW estimated for Ler (black circle) was 5.55, corresponding to ~355 kDa (24.8 Ler units).

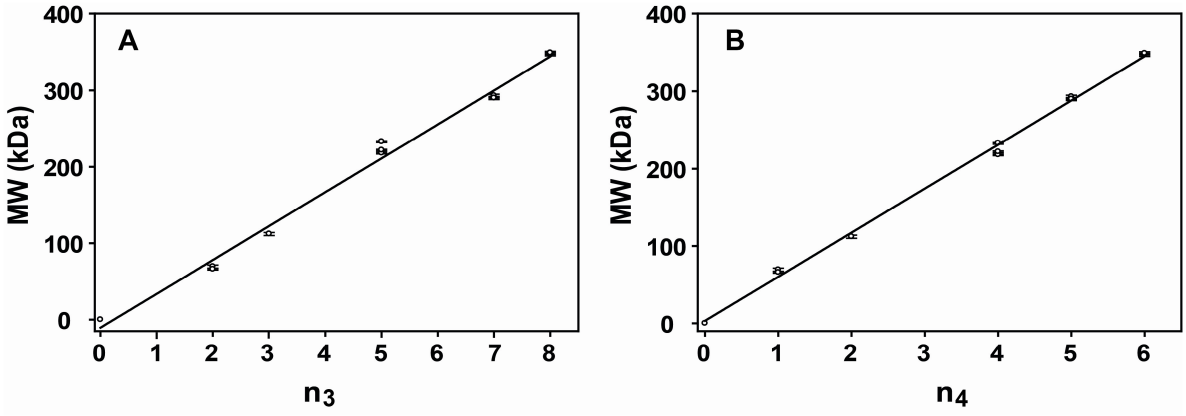

Fig. S4. Linear correlation of apparent molecular weight values assuming alternative helical Ler oligomers. The range of molecular weights observed can be explained as full turns (n_3, n_4) of helices containing three (**A**) or four (**B**) monomers per turn, respectively. In the best fit lines, points corresponding to 3, 12 and 18 monomers (**A**) or 12 monomers (**B**) are not observed. The slope is 44.3 ± 1.3 kDa (R^2 = 0.99) and 56.9 ± 1.0 kDa (R^2 = 0.99), corresponding to 3.1 and 3.98 Ler units, respectively.

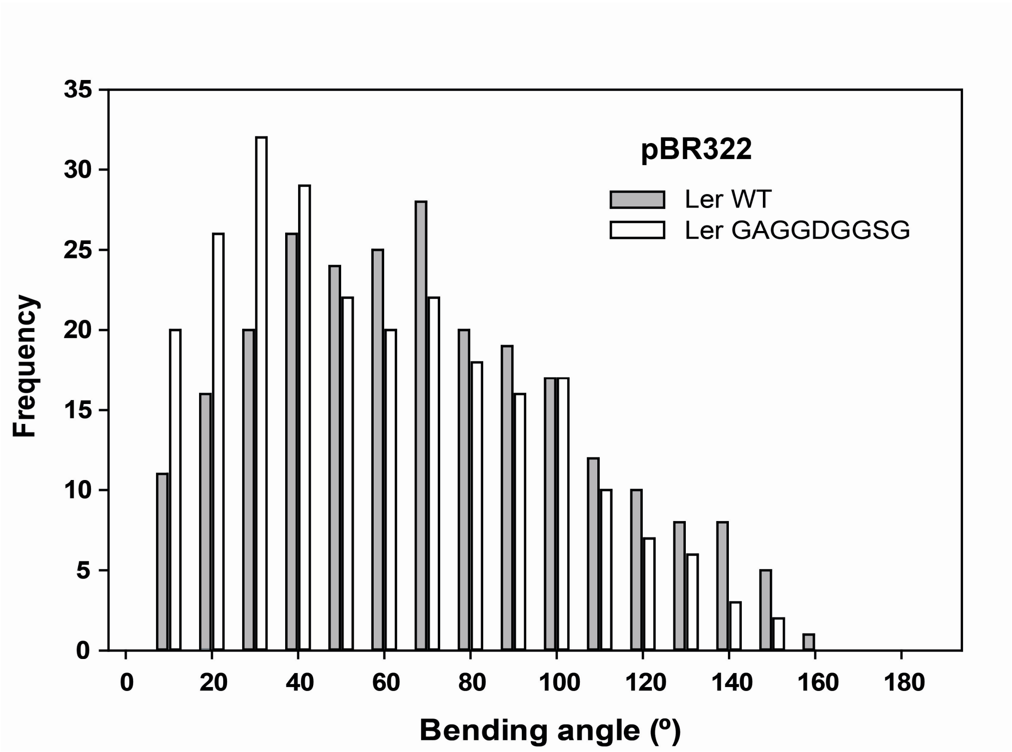

Fig. S5. A Ler mutant with an extended connector linking the DNA-binding and the oligomerization domains induces smaller DNA bending. A nine amino acid sequence, GAGGDGGSG, was inserted between positions K73 and G74 of Ler, just before the start of the structured C-terminal domain (position 76). The inserted segment is predicted to be highly flexible. The histogram of the bending-angle distributions induced by Ler WT (250 complexes) and the Ler mutant (250 complexes) in pBR322 molecules is shown. The two distributions were significantly different (P<0.003) and the mutant induced lower bending-angles.

Table S1. AFM volumes of Ler in the absence or presence of DNA (Lee1192).

Ler	DNA	Volume (nm^3)[a]	Apparent M_W (kDa)[b]	Ler units[c]
Isolated	-	166 ± 8	69 ± 2	4.8
Isolated	-	790 ± 3	218 ± 1	15.2
Isolated	-	1107 ± 9	293 ± 2	20.5
DNA-bound	Lee1192	853 ± 4	233 ± 1	16.3
DNA-bound	Lee1192	1093 ± 3	290 ± 1	20.3
DNA-bound	Lee1192	1341 ± 9	349 ± 2	24.4
Free	Lee1192	153 ± 3	66 ± 1	4.6
Free	Lee1192	344 ± 5	112 ± 1	7.8
Free	Lee1192	806 ± 3	222 ± 1	15.5
Free	Lee1192	1092 ± 5	290 ± 1	20.3
Free	Lee1192	1333 ± 8	347 ± 2	24.3

[a]Values represent the mean (± the standard error of the mean) of the Gaussian fit to the distributions reported in Figure 5.
[b]The apparent molecular weight of Ler particles was estimated from the calibration obtained by measuring a set of reference proteins of known molecular weight (Figure S1).
[c]Each Ler unit has a predicted mass of 14.3 kDa.
